# Evaluation of Cytocompatibility and Anti-Inflammatory Activity of Carboxyxanthones Selected by In Silico Studies

**DOI:** 10.3390/ijms27010110

**Published:** 2025-12-22

**Authors:** Ricardo F. Pereira, Catarina Amoedo-Leite, Sara Gimondi, Sara F. Vieira, João Handel, Andreia Palmeira, Maria Elizabeth Tiritan, Madalena M. M. Pinto, Nuno M. Neves, Helena Ferreira, Carla Fernandes

**Affiliations:** 1 Laboratório de Química Orgânica e Farmacêutica, Departamento de Ciências Químicas, Faculdade de Farmácia, Universidade do Porto, Rua Jorge Viterbo Ferreira n° 228, 4050-313 Porto, Portugal; ricardojose.fariapereira@unifi.it (R.F.P.); catarina.leite@mcb.uu.se (C.A.-L.); up201107677@edu.ff.up.pt (J.H.); apalmeira@ff.up.pt (A.P.); beth@ff.up.pt (M.E.T.); madalenakijjoa@gmail.com (M.M.M.P.); 2 3B’s Research Group, I3BS—Research Institute on Biomaterials, Biodegradables and Biomimetics, University of Minho, Headquarters of the European Institute of Excellence on Tissue Engineering and Regenerative Medicine, AvePark, Parque de Ciência e Tecnologia, Rua Ave 1, Edifício 1 (Sede), Barco, 4805-694 Guimarães, Portugal; sara.gimondi@i3bs.uminho.pt (S.G.); sara.vieira@i3bs.uminho.pt (S.F.V.); nuno@i3bs.uminho.pt (N.M.N.); 3 ICVS/3B’s, PT Government Associate Laboratory, 4806-909 Braga/Guimarães, Portugal; 4 CIIMAR/CIMAR LA, Interdisciplinary Centre of Marine and Environmental Research, Universidade do Porto, Terminal de Cruzeiros do Porto de Leixões, Avenida General Norton de Matos, s/n, 4450-208 Matosinhos, Portugal; 5 UCIBIO, Applied Molecular Biosciences Unit, Translational Toxicology Research Laboratory, University Institute of Health Sciences (1H-TOXRUN, IUCS-CESPU), 4585-116 Gandra, Portugal; 6 Associate Laboratory i4HB—Institute for Health and Bioeconomy, University Institute of Health Sciences—CESPU, 4585-116 Gandra, Portugal

**Keywords:** xanthone derivatives, carboxyxanthones, diaryl ether intermediates, anti-inflammatory activity, lipopolysaccharide-stimulated macrophages, interleukin 6, cyclooxygenase-2, prostaglandin E_2_, docking, structure–activity relationship (SAR) analyses, drug discovery

## Abstract

Carboxyxanthones containing carboxylic acid groups linked to lipophilic aromatic rings resemble the key pharmacophoric features of many nonsteroidal anti-inflammatory drugs (NSAIDs). This structural similarity makes them attractive scaffolds for the development of new anti-inflammatory agents. This study describes the production, cytocompatibility, and anti-inflammatory potential of ten carboxyxanthones (**1**–**10**) and two intermediates (**11**–**12**) by evaluating their effects on key pro-inflammatory mediators, namely interleukin 6 (IL-6) and prostaglandin E2 (PGE_2_). As these compounds are produced by distinct mechanisms, their multi-target potential will be evaluated. Carboxyxanthones were obtained by multi-step pathways using different synthetic approaches through classical benzophenone or diaryl ether intermediates synthesis followed by intramolecular acylation. To the best of our knowledge, the synthesis of carboxyxanthones **3** and **5** is described herein for the first time. All tested compounds were cytocompatible with lipopolysaccharide (LPS)-stimulated macrophages. The most notable carboxyxanthones were **3**, **4**, **7**, and **8**, which were able to significantly reduce IL-6 production by approximately 60%. Molecular docking simulations between compounds **1**–**12** and cyclooxygenase-2 were conducted to characterize the structural features underlying molecular recognition, and to identify the most promising candidates for subsequent PGE_2_ assays. Carboxyxanthones **3**, **5**, and **6**, as well as intermediate **12**, were predicted to be the best. In the human in vitro inflammation model used, carboxyxanthone **6** exhibited the most potent and consistent inhibitory effect on PGE_2_ production. At the highest concentration tested (100 µM), it presented an efficacy comparable to that of celecoxib. Carboxyxanthones **3** and **5** demonstrated a biphasic effect, decreasing and increasing PGE_2_ production at lower (5, 12.5, and 25 µM) and higher (50 and 100 µM) concentrations, respectively. These results highlight the potential of carboxyxanthones as promising modulators of inflammatory pathways, paving the way for further studies aimed at elucidating their mechanisms of action, optimizing structural features, and assessing their safety and therapeutic potential in relevant disease models.

## 1. Introduction

Inflammation is a fundamental biological response for host defense and tissue repair. However, when dysregulated or persistent, inflammation can become pathological, contributing to the onset and progression of numerous chronic conditions, including arthritic and autoimmune diseases [1,2]. In unresolved inflammatory states, activated immune cells release high levels of inflammatory mediators, such as reactive oxygen and nitrogen species (ROS/RNS, e.g., peroxyl radical—ROO^●^, and nitric oxide—^●^NO), cytokines (e.g., interleukin—IL-6 and tumor necrosis factor—TNF-α), chemokines (e.g., IL-8 and monocyte chemoattractant protein—MCP-1), and eicosanoids (e.g., prostaglandin E2—PGE_2_, thromboxane—TXA2 and leukotriene—LTB4) [3,4,5]. These mediators orchestrate complex signaling networks that drive tissue degeneration, fibrosis, and loss of physiological function. Given its central role in a wide range of pathologies, the effective control and resolution of chronic inflammation is of critical therapeutic importance [6,7].

Current treatment strategies involve a broad range of pharmacological agents, including nonsteroidal anti-inflammatory drugs (NSAIDs, e.g., ibuprofen and celecoxib), glucocorticoids (e.g., dexamethasone and betamethasone), conventional synthetic disease-modifying antirheumatic drugs (csDMARDs, e.g., methotrexate and sulfasalazine), biologic DMARDs (bDMARDs, e.g., infliximab and rituximab), and targeted synthetic DMARDs (tsDMARDs, e.g., tofacitinib and filgotinib). These agents reduce inflammation through various mechanisms [3]. For instance, NSAIDs that are the most commonly used for managing inflammatory conditions [8], exert their effects primarily through the inhibition of cyclooxygenase-2 (COX-2) [9]. The inhibition of this inducible enzyme reduces the production of pro-inflammatory mediators, such as PGE_2_. However, they can also inhibit COX-1, a constitutively expressed enzyme essential for the regulation of homeostatic functions [10]. This non-selective inhibition is associated with diverse side effects, including gastrointestinal irritation, renal impairment, and increased cardiovascular risk [11,12]. To address these limitations, selective COX-2 inhibitors were developed to minimize COX-1-related side effects. Nonetheless, several of these selective agents were withdrawn from the market due to their association with serious cardiovascular events [10]. Currently, celecoxib is the only selective COX-2 inhibitor available for clinical use by both the European Medicines Agency (EMA) and the U.S. Food and Drug Administration (FDA) [11]. As a result, the majority of patients receive non-selective NSAIDs.

Given the serious side effects, limited long-term efficacy, and/or high costs of the current therapies, there is a growing demand for novel therapeutic agents that provide effective anti-inflammatory action with improved safety profiles. As such, identifying new molecular scaffolds and exploring bioactive compounds with multi-target properties represent key strategies in the search for safer and more cost-effective therapies [13].

Natural products offer a valuable source of therapeutic candidates due to their structural diversity and ability to modulate multiple biological pathways [3]. Among these, xanthones have attracted significant attention due to their wide range of biological activities, including anti-inflammatory, antioxidant, and antitumoral effects [14,15]. The xanthone scaffold is considered a privileged structure in medicinal chemistry, offering considerable flexibility for chemical modification and optimization. The synthesis of xanthone derivatives enables the development of novel compounds not present in nature [16]. The diversity of substituents and substitution patterns that can be introduced onto the xanthone core can contribute to the development of compounds with enhanced pharmacological potential and diverse biological activities [17]. Thus, the exploration and synthesis of xanthone derivatives represent a promising avenue for the discovery of more effective therapeutic agents for various diseases, including chronic inflammatory disorders [18].

Many xanthone derivatives have been reported to exhibit anti-inflammatory properties across different targets and through diverse mechanisms of action [18]. For example, the naturally occurring xanthone derivatives β-mangostin [19], 1,3,6,7-tetrahydroxy-8-prenylxanthone [20], and garcinoxanghones B and C [21] were shown to reduce NO production in lipopolysaccharide (LPS)-stimulated macrophages while cudratricusxanthone A was found to induce heme oxygenase-1 expression [22]. Several synthetic chiral derivatives were also reported to inhibit both COX-1 and COX-2 [15]. In addition to these enzymes, 6-dihydroxy-5-methoxy-4′,5′-dihydro-4′,4′,5′-trimethylfurano-(2′,3′:3,4)-xanthone was found to inhibit 5-lipoxygenase [23]. Furthermore, the xanthone derivative ellidifolin inhibited the production of the pro-inflammatory cytokines IL-6 and TNF-*α* in LPS-stimulated macrophages, as well as the production of PGE_2_ [24].

In particular, carboxyxanthones, which contain a carboxylic acid group and lipophilic aromatic rings, resemble the pharmacophore of many NSAIDs [25]. These features make them attractive candidates for the development of new anti-inflammatory drugs [26]. Moreover, carboxyxanthones were reported to decrease the concentration of the pro-inflammatory cytokine IL-6 on LPS-stimulated macrophages [27]. Chiral derivatives of carboxyxanthones have also been effective in reducing IL-6 production [28].

**Figure 1:**
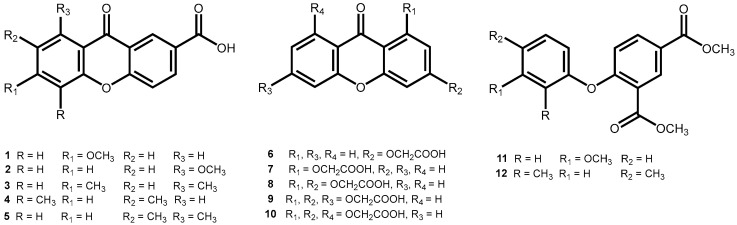
Chemical structures of carboxyxanthones (**1**–**10**) and intermediates (**11**–**12**).

This study describes the synthesis and investigates the cytocompatibility and anti-inflammatory potential of a series of carboxyxanthones and intermediates (Figure 1). After chemical characterization, the anti-inflammatory activity was evaluated by their effects on the production of key pro-inflammatory mediators, namely IL-6 and PGE_2_. Since the reduction in IL-6 production involves the inhibition of signaling pathways, such as nuclear factor-kappa B (NF-κB) [29], and the suppression of PGE_2_ requires inhibition of the COX-2 enzyme [9], the compounds were analyzed for their potential multi-target activity. Molecular modeling studies using the docking technique were conducted to elucidate the interactions of carboxyxanthones and intermediates with the COX-2 active site. This approach enabled the characterization of the structural features underlying molecular recognition, and the identification of the most promising compounds for subsequent PGE_2_ assays. Furthermore, structure–activity relationship (SAR) analyses were performed to support the rational design of safer and more effective anti-inflammatory agents.

## 2. Results and Discussion

### 2.1. Synthesis of Carboxyxanthones

To the best of our knowledge, the synthesis of carboxyxanthones **3** and **5** was described herein for the first time. The remaining carboxyxanthones (**1**–**2**, **4**, **6**–**10**) were synthesized according to previously reported procedures [30,31,32,33].

**Scheme 1:**
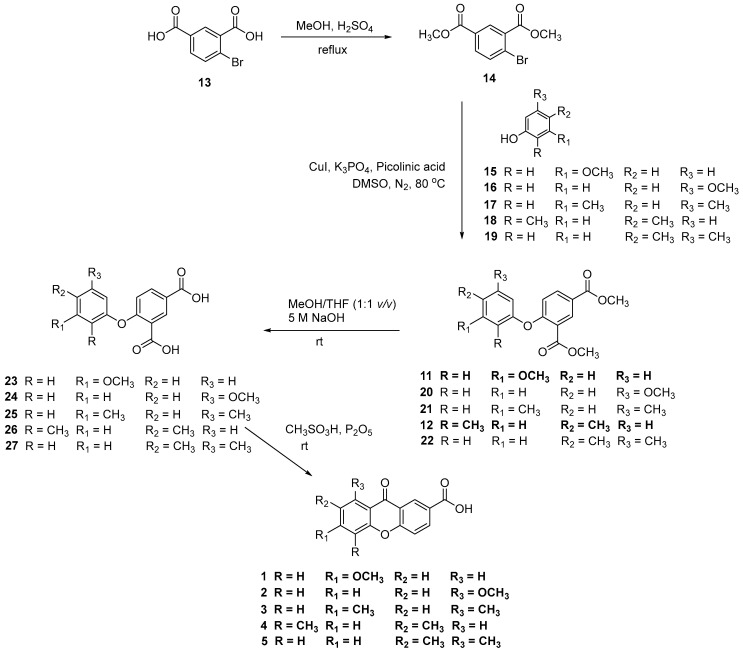
Total synthesis of carboxyxanthones **1**–**5**.

The carboxyxanthones **1**–**5** were obtained through a multi-step pathway, illustrated in Scheme 1. A modified Ullmann reaction was used to produce diaryl ether intermediates (**11**–**12** and **20**–**22**) from the aryl bromide **14** and an appropriate phenol (**15**–**19**). The catalyst system composed of CuI and picolinic acid in combination with K_3_PO_4_/dimethyl sulfoxide (DMSO) proved to be efficient [34]. The aryl bromide **14** was previously prepared from the corresponding carboxylic acid **13** by Fisher esterification. Notably, the yields achieved for the diaryl ether intermediates of the new carboxyxanthones, namely intermediates **21** and **22** from carboxyxanthones **3** and **5**, respectively, exceeded 80% in both cases. In the next step, the hydrolysis of the ester groups of the diaryl ether intermediates (**11**–**12** and **20**–**22**), in the presence of an aqueous base, was performed to provide the respective carboxylic acids (**23**–**27**). Then, through further intramolecular acylation, using phosphorus pentoxide and methane sulfonic acid (Eaton’s reagent), carboxyxanthones **1**–**5** were obtained (Scheme 1). The new synthetized carboxyxanthones **3** and **5** were obtained in yields of 52% and 69%, respectively.

It was observed that the intramolecular acylation of carboxyxanthones **1**, **2**, and **5** led to the formation of isomeric carboxyxanthones in varying yields. The isomers were separated through Fisher esterification. Purification was achieved by filtration considering their great solubility differences in methanol (ester derivatives of **1** and **2** [33]) or by column chromatography (ester derivatives of **5**). After separation, the ester derivatives of **1**, **2**, and **5** were hydrolyzed in an alkaline medium, yielding the carboxyxanthones **1**, **2**, and **5**.

**Scheme 2:**
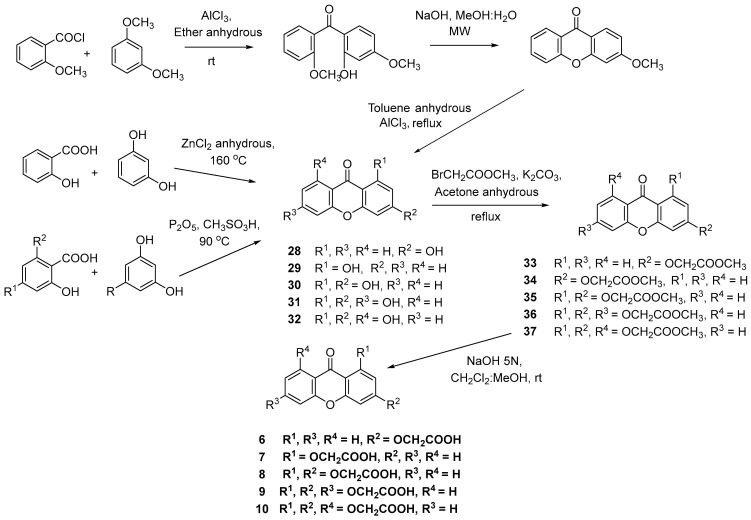
Total synthesis of carboxyxanthones **6**–**10**.

Carboxyxanthones **6**–**10** were obtained by multi-step pathways as illustrated in Scheme 2. First, various hydroxylated xanthones (**28**–**32**) were synthesized as precursors, using different synthetic approaches. The hydroxylated xanthone **28** was obtained by a classical benzophenone intermediate synthesis followed by intramolecular acylation under microwave irradiation and demethylation [31]. Hydroxylated xanthones **29**–**32** were obtained through the condensation of appropriate building blocks, in the presence of the Lewis acid ZnCl_2_ (**29**) [35] or P_2_O_5_/CH_3_SO_3_H (Eaton’s reagent) (**30**–**32**) [36,37,38]. The hydroxylated xanthones (**28**–**32**) were then oxidized to afford the acetate intermediates (**33**–**37**). A Williamson ether synthesis was performed using an alkyl halide containing an ester group (BrCH_2_COOCH_3_). In the last step, an alkaline hydrolysis of the respective esters of **33**–**37** afforded the carboxyxanthones **6**–**10** (Scheme 2).

The structures of the synthetized carboxyxanthones (**1**–**10**) and all intermediates were elucidated by nuclear magnetic resonance (NMR), Fourier-transform infrared spectroscopy (FTIR) and high-resolution mass spectrometry (HRMS). The ^1^H NMR, ^13^C NMR spectra and HRMS data for the new carboxyxanthones (**3** and **5**) are shown in Supplementary Materials (Figures S1–S4).

The synthetic methodologies used herein proved to be efficient and broadly applicable for obtaining diverse carboxyxanthones in good yields. Furthermore, the procedures can be readily scaled up to produce sufficient quantities for further biological assays.

### 2.2. Cytocompatibility

**Figure 2:**
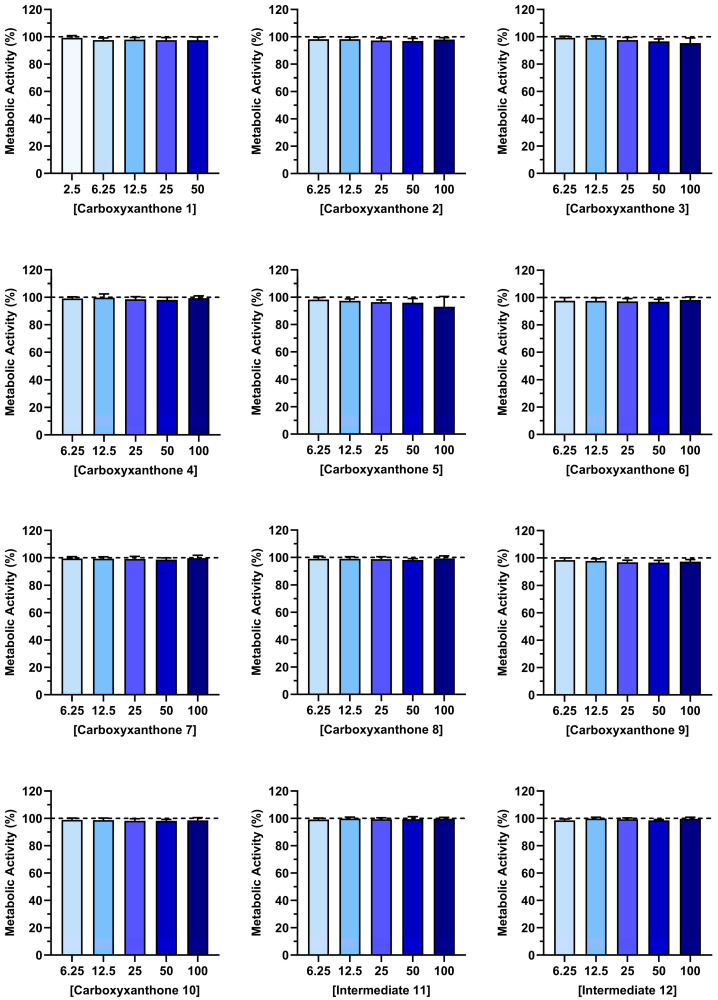
Metabolic activity of LPS-stimulated macrophages cultured in the presence of different concentrations of carboxyxanthones (**1**–**10**) and intermediates (**11**–**12**) for 24 h of culture. The dotted line represents the nontreated condition (0 µM). Data were obtained from experiments performed with three independent biological samples, each analyzed in technical triplicates (total of 9 measurements per condition). Results are presented as mean ± SD. There are no statistically significant differences in comparison to the negative control.

**Figure 3:**
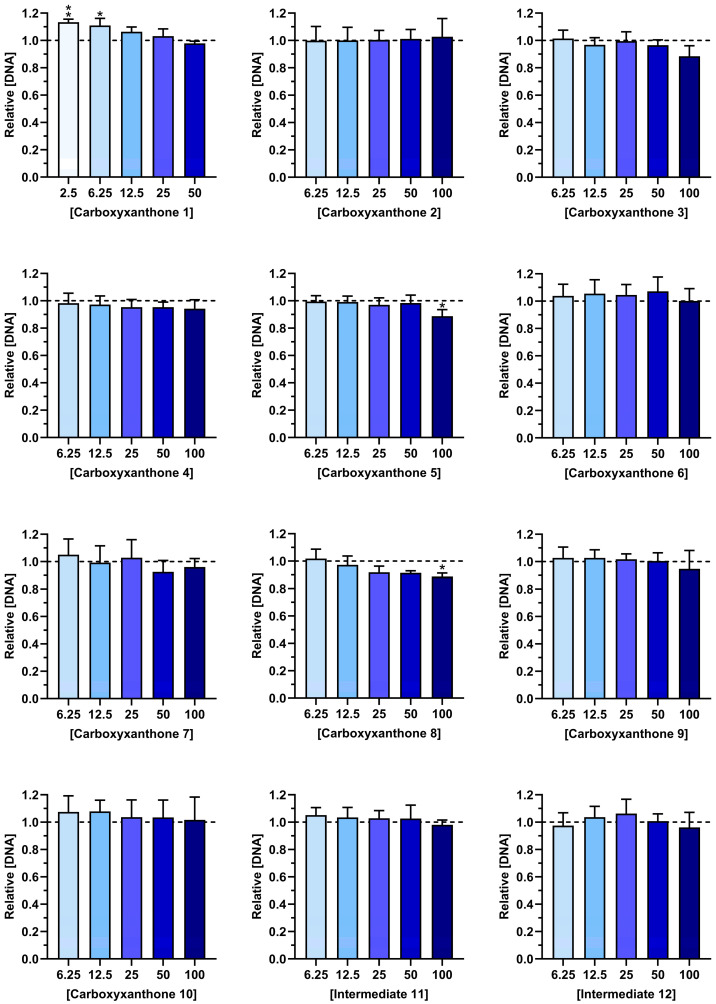
Relative DNA concentration of LPS-stimulated macrophages cultured in the presence of different concentrations of carboxyxanthones (**1**–**10**) and intermediates (**11**–**12**) for 24 h of culture. The dotted line represents the nontreated condition (0 µM). Data were obtained from experiments performed with three independent biological samples, each analyzed in technical triplicates (total of 9 measurements per condition). Results are presented as mean ± SD. Statistically significant differences are * (*p* < 0.05) and ** (*p* < 0.01) in comparison to the negative control (0 µM) for each different tested compound.

Before evaluating the therapeutic potential, it is essential to assess the cytocompatibility of the tested compounds. Determining that these compounds do not negatively affect cell viability is a critical step in pharmaceutical development, particularly when targeting immune cells, such as macrophages [39]. The cytocompatibility of the synthesized compounds (**1**–**12**) was evaluated using activated macrophages. Figure 2 and Figure 3 show, respectively, the metabolic activity and DNA content of LPS-stimulated macrophages, after exposure to different concentrations of the tested compounds over a period of 24 h. The synthetic compounds did not compromise the metabolic activity of LPS-stimulated macrophages at any of the studied concentrations (6.25–100 µM) (Figure 2). Moreover, the DNA content of macrophages was preserved in the presence of the different compounds. Only carboxyxanthones **5** and **8**, at the highest concentration (100 µM), significantly reduced the DNA content of LPS-stimulated macrophages (Figure 3). However, according to ISO 10993-5 [40], they can be considered cytocompatible since cell viability and DNA amount remained above 70%. Overall, the observed cytocompatible concentration range supports the potential of these compounds for further pharmaceutical development.

### 2.3. IL-6 Quantification

**Figure 4:**
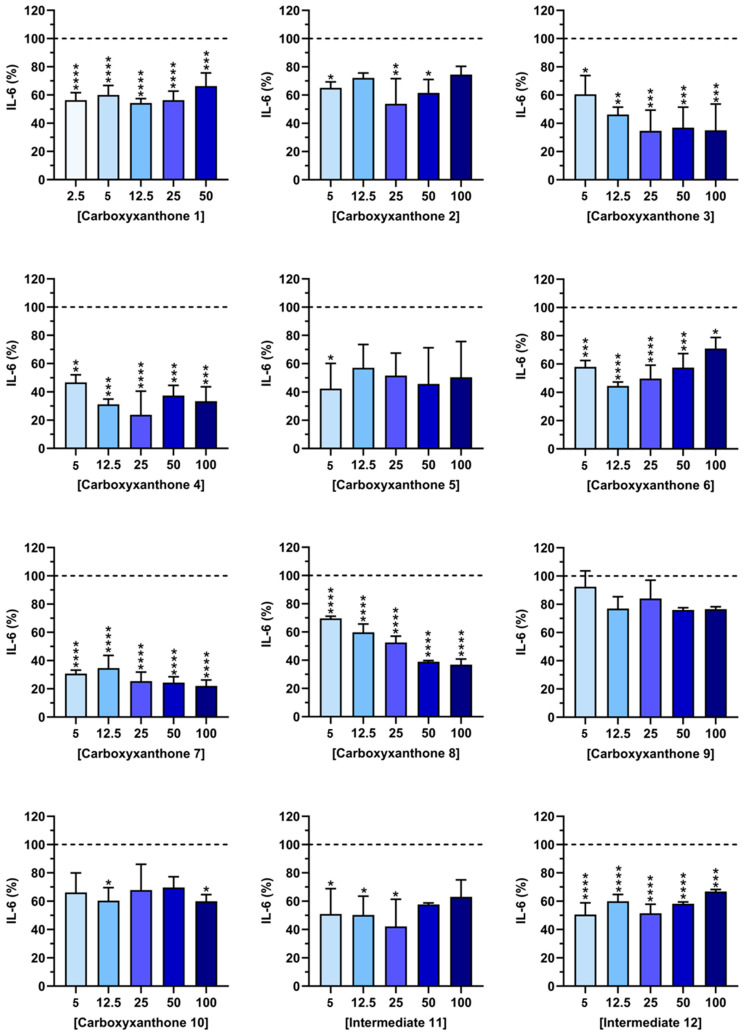
IL-6 production by LPS-stimulated macrophages cultured for 24 h in the presence of increasing concentrations of carboxyxanthones (**1**–**10**) and intermediates (**11**–**12**). IL-6 levels were normalized to DNA content for each condition and expressed as a percentage relative to the nontreated control (0 µM; dotted line). Data were obtained from experiments performed with three independent biological samples, each analyzed in technical triplicates (total of 9 measurements per condition). Results are presented as mean ± SD. Statistical analysis was conducted using one-way ANOVA with multiple comparisons against the 0 µM control for each compound, followed by Dunnett’s post hoc test. Significance levels are indicated as follows: * *p* < 0.05, ** *p* < 0.01, *** *p* < 0.001, **** *p* < 0.0001. The corresponding full numeric data (means ± SDs) and sample sizes (n) are provided in Table S1.

Inflammation plays a central role in the pathogenesis of numerous diseases, and the inhibition of proinflammatory cytokine production remains a key therapeutic strategy [41]. Among these mediators, IL-6 is particularly relevant due to its involvement in both acute and chronic inflammatory responses [3]. To investigate their anti-inflammatory properties, the effects of carboxyxanthones **1**–**10** and intermediates **11**–**12** on IL-6 secretion were analyzed in LPS-stimulated macrophages (Figure 4). Non-stimulated macrophages produced only basal levels of IL-6 (0.19 ± 0.35%), whereas LPS stimulation successfully induced a robust inflammatory response. Dexamethasone (10 µM), used as a positive control, markedly reduced IL-6 production by 89.06 ± 10.45%.

As shown in Figure 4, several tested compounds significantly decreased IL-6 levels. Carboxyxanthones **3**, **4**, **7**, and **8** displayed the most pronounced inhibitory effects (≈60% IL-6 reduction), with significant reductions observed at a concentration of 25 µM. Carboxyxanthones **1**, and **6**, and intermediate **12,** also reduced IL-6 production, although to a lesser extent (≈50%). Conversely, carboxyxanthones **2**, **5**, **9**, and **10**, and intermediate **11** exhibited minimal or no statistically significant activity (≈45%). The differences in anti-inflammatory activity among the tested compounds can be related to the type and position of substituents attached to the xanthone scaffold. Carboxyxanthones **3**, **4**, **7**, and **8**, which showed the strongest inhibition of IL-6 production, shared structural features that may enhance electronic delocalization and facilitate interactions with biological targets involved in inflammatory signaling. In particular, the dimethyl-substituted derivatives (carboxyxanthones **3** and **4**) suggest that methyl groups at positions 6, 8, or 5, 7 may increase the lipophilicity of the molecule, improving its cell permeability and possibly stabilizing the xanthone core in its active conformation. The presence of ether linkages and carboxyl groups in carboxyxanthones **7** and **8** may contribute to increased polarity and hydrogen-bonding capacity, enabling stronger interactions with molecular targets, such as enzymes or receptors involved in cytokine regulation. Notably, the oxyacetic acid substituent in carboxyxanthone **7** showed greater inhibitory potency than the bis-oxyacetic acids present in carboxyxanthone **8**. This suggests that a single oxyacetic substituent at position 1 better balances polarity and steric effects for enhanced activity. The less pronounced activity of carboxyxanthones **1** and **6** could be attributed to the presence of only a single substituent or to the steric limitations that hinder interaction with the target site. Carboxyxanthones **2**, **5**, **9**, and **10**, and intermediate **11** further support that excessive substitution or substitution at non-optimal ring positions can reduce electronic conjugation and compromise biological activity.

Collectively, these results indicate that the anti-inflammatory potential of the tested compounds depends strongly on substitution pattern and functional group position. Small hydrophobic groups, such as methyl substituents and moderately polar side chains (oxyacetic acids), appear to favor IL-6 inhibition, likely by optimizing the balance between molecular lipophilicity and hydrogen-bonding ability. This SAR analysis highlights the importance of fine-tuning substituent position and polarity to design more effective xanthone-based anti-inflammatory agents.

### 2.4. Structure-Based Virtual Screening

The process of drug discovery is inherently complex, demanding substantial time, financial investment, and multidisciplinary effort. The integration of computational tools with conventional chemical and biological assays has emerged as a powerful strategy to enhance the efficiency of this process. Indeed, it increases the success rate of drug screening and reduces the empirical nature of early-stage research. In silico approaches significantly accelerate and refine various phases of drug discovery, including hit identification. One of the most prominent and effective structure-based virtual screening tools is the molecular docking [42,43].

This study aimed to assess the anti-inflammatory potential of carboxyxanthones **1**–**10** and the intermediates **11**–**12**, focusing on their effects not only on IL-6 but also on PGE_2_ production. As PGE_2_ suppression depends on COX-2 inhibition [9], molecular docking simulations between compounds **1**–**12** and COX-2 were conducted to identify the most promising candidates for subsequent PGE_2_ assays. COX-1 was also included for comparative purposes and to predict safer compounds. The binding interactions of synthesized compounds with the COX-2 active site were further analyzed to elucidate the structural features underlying molecular recognition.

The poses obtained on the redocking protocol reproduced the crystallographic ligand orientations and interactions described in the literature (Figure S5), confirming that the docking protocol, parameterization, and grid box dimensions were suitable for recovering biologically relevant binding poses. This redocking procedure validates the reliability of the docking pipeline applied in this study.

**Figure 5:**
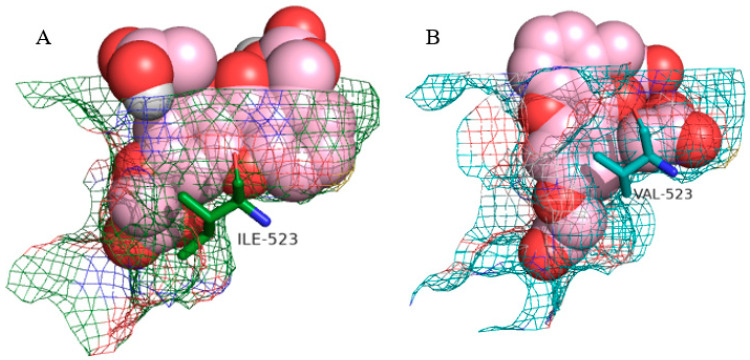
Carboxyxanthone **10** molecular conformational changes in COX-1 (**A**) and COX-2 (**B**). COX-1 is depicted in green, while COX-2 is depicted in blue. The ligand and the enzyme binding site are represented as spheres and a mesh, respectively.

**Figure 6:**
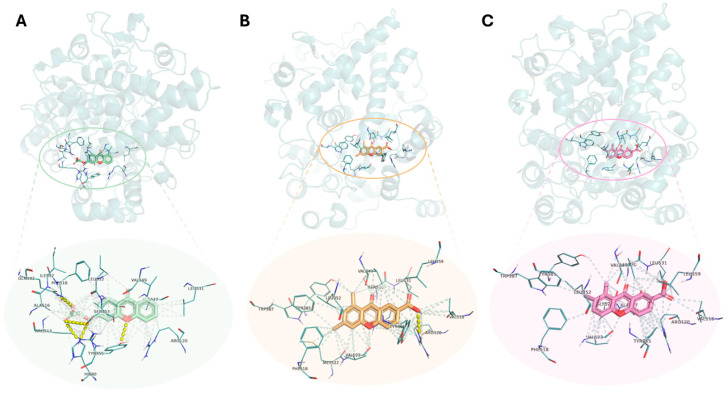
Docking poses of carboxyxanthone **3** (**A**), carboxyxanthone **5** (**B**), and carboxyxanthone **6** (**C**) in the COX-2 active site. Carboxyxanthone **3**, **5** and **6** are depicted in green, orange and pink, respectively. The residues involved in hydrogen-bonding, polar, and hydrophobic interactions are represented as lines. Yellow dashes represent hydrogen-bonding interactions. The gray dashes represent other possible interactions between the ligands and the enzyme.

Non-selective NSAIDs, namely naproxen, piroxicam, diclofenac, indomethacin, and (*S*)-ibuprofen, and selective COX-2 inhibitors, such as celecoxib and valdecoxib [44], were used as positive controls. The binding affinity between both enzymes and the controls and tested compounds was evaluated by the binding free energy approximation (∆G, kcal/mol). A lower score indicates a greater affinity of the compound for the target enzyme [45]. For each ligand, the top-ranked pose (lowest ΔG) was extracted, and results were organized in Table 1, which lists: number of polar interactions, interacting functional groups, interacting amino acid residues and bond lengths, and docking scores for COX-1 and COX-2. Graphical representations (Figure 5 and Figure 6) further illustrate the docking poses and interaction patterns of representative compounds, enabling direct visual comparison between the carboxyxanthones and the positive controls.

To compare the docking performance of the synthesized carboxyxanthones with the reference NSAIDs, the predicted binding free energies (ΔG, kcal/mol) were analyzed. Mathematically, we compared the top-ranked docking score of each compound with the scores obtained for the positive controls (celecoxib, diclofenac, indomethacin, naproxen, piroxicam, and (*S*)-ibuprofen) using a direct numerical comparison of ΔG values in COX-1 and COX-2 enzymes. This included evaluating the range and distribution of ΔG values and identifying compounds whose predicted affinities were equal to or more favorable than the reference inhibitors. Graphically, these comparisons were illustrated through 3D interaction diagrams, where the binding poses and key residue interactions of the test compounds were visually contrasted with those of the controls. Together, these mathematical and graphical analyses enabled a clear and reproducible assessment of how the synthesized molecules perform relative to clinically used COX inhibitors.

All positive controls showed negative binding energy values (Table 1). The tested compounds successfully docked into the active site of COX-1 and COX-2, obtaining docking scores similar to or, in some cases, even more negative than the ones obtained for the positive controls. This indicates that the predicted affinity of several test compounds is within the range of established anti-inflammatory drugs. For example, celecoxib and valdecoxib, exhibited −10.0 and −9.0 kcal/mol, respectively, and both diclofenac and indomethacin presented −8.3 kcal/mol for COX-2 inhibition. Among the tested compounds, the carboxyxanthones **3** (−9.0 kcal/mol), **5** (−9.0 kcal/mol), and **6** (−9.4 kcal/mol), and the intermediate **12** (−9.1 kcal/mol) exhibited the lowest binding free energies for COX-2 and, therefore, the highest binding affinities. Furthermore, for these compounds, a relevant difference was observed between their binding affinities toward COX-2 and COX-1. In particular, intermediate **12** displayed a binding free energy of −7.6 kcal/mol for COX-1, whereas the corresponding value for COX-2 was −9.1 kcal/mol. This finding is noteworthy, as it highlights a desirable preferential binding to COX-2, thereby indicating a potential reduction in, e.g., gastrointestinal adverse effects commonly associated with COX-1 inhibition.

Regarding the amino acid residues, Arg 120, present in the active site of both COX-1 and COX-2 isoforms, is a residue with which most inhibitors establish interactions. Arg 120 stands out from all amino acids as this residue establishes polar interactions with several ligands in both enzymes. Since Arg 120 is a charged amino acid, an ionic interaction between the carboxylate anion of the inhibitor and the guanidinium cation of the residue is established [46]. This interaction is observed between the (*S*)-ibuprofen and the COX-1 enzyme, and between the carboxyxanthones **1** and **3** and the COX-2 enzyme. This interaction is also present in the substrate-enzyme binding mechanism, when Arg 120 binds to the carboxylate group of the arachidonic acid [47]. Other amino acid residues also contribute to polar interactions with ligands in both isoforms, such as Ser 530 and Tyr 355, which particularly participate in hydrogen bond formation. In contrast, certain residues, including His 90 and Gln 192, predominantly establish hydrogen bond interactions with the tested ligands in the COX-2 enzyme.

In addition to hydrogen bond interactions, other interactions play a relevant role in molecular recognition, including the permanent dipole and hydrophobic interactions within the hydrophobic channel of the enzyme’s active site. These interactions contribute to ligand stabilization and enhanced binding affinity [48]. As an example, Table 2 summarizes all the interactions observed by the most promising tested compounds (**3**, **5**, **6**, and **12**) and celecoxib within the COX-2 active site. The amino acid residues involved are highlighted.

Among the amino acid residues that interact with the tested molecules, Tyr 385 is particularly important. It assumes the role of the catalytic residue by catalyzing the oxygenation reaction. Therefore, binding a ligand to this residue is favorable to the inhibition of the prostaglandin biosynthesis mechanism [47]. Arg 513, Ala 516, and Val 523, as COX-2 exclusive amino acid residues, have a relevant role in ligand–enzyme binding. For instance, the substitution of His 513 by an arginine residue in the COX-2 isoform introduces a stable positive charge in the chemical environment of the hydrophobic main chain. This enables selective interactions with polar groups [47,49]. Similarly, Ala 516 (Ser 516 in COX-1) is another residue that might contribute to specific interactions with the COX-2 enzyme [48]. The Val 523 amino acid residue, as described earlier, is one of the most important residues in COX-2. By creating the hydrophilic side-pocket and widening the hydrophobic channel, this residue leads to conformational changes in the ligands that can heavily impact their binding affinity. This can be observed by comparing the molecular conformation of carboxyxanthone **10** in COX-1 and COX-2, as illustrated in Figure 5.

To illustrate the results obtained in the docking studies, representative examples of the most stable docked conformation of carboxyxanthone **3**, carboxyxanthone **5**, and carboxyxanthone **6** are illustrated in Figure 6. These were some of the tested molecules with higher binding energy within the COX-2 active site. The hydrogen bonds are exposed, as well as the surface equivalence of the polar and hydrophobic interactions.

Overall, both the statistical (ΔG comparisons) and graphical analyses (Figures of binding poses and interactions) demonstrate that the tested carboxyxanthones exhibit binding affinities and interaction profiles comparable to known COX inhibitors, supporting their relevance as potential anti-inflammatory candidates.

Although the docking simulations rely on static crystal structures of COX-1 and COX-2 and therefore do not capture protein flexibility or induced-fit effects, and although they do not account for assay conditions such as the presence of 0.2% DMSO or potential off-target interactions beyond COX isoforms, these limitations are inherent to standard and commonly used docking protocols. However, the use of high-quality crystal structures, consistent methodology, and concordant interaction patterns with well-established NSAIDs support the robustness of our predictions. Thus, while the in silico results should be interpreted as qualitative mechanistic insights rather than absolute measures of affinity, they remain reliable and informative for guiding structure-activity trends and prioritizing the most promising carboxyxanthones for biological evaluation. Thus, the docking studies allowed us to predict the potential anti-inflammatory activity of the synthesized compounds by analyzing their enzyme–ligand binding affinities, preferred conformations, and the interactions with key residues essential to the COX-ligand binding. Future studies will include determining IC_50_ values for both COX-1 and COX-2 using highly purified COX-1 and COX-2 enzymes. This in silico data was used to select the most promising candidates for subsequent PGE_2_ assays, specifically the carboxyxanthones **3**, **5**, and **6** and the intermediate **12**.

### 2.5. PGE_2_ Quantification

PGE_2_ is a key pro-inflammatory lipid mediator synthesized via the COX-2 pathway. It plays a critical role in the progression of inflammatory responses, contributing to pain, fever, and tissue injury [3].

To evaluate the ability of carboxyxanthones **3**, **5**, and **6** and intermediate **12** to modulate PGE_2_ production, a human in vitro inflammatory model was used. In this model, macrophages were stimulated with LPS to mimic a pro-inflammatory phenotype.

**Figure 7:**
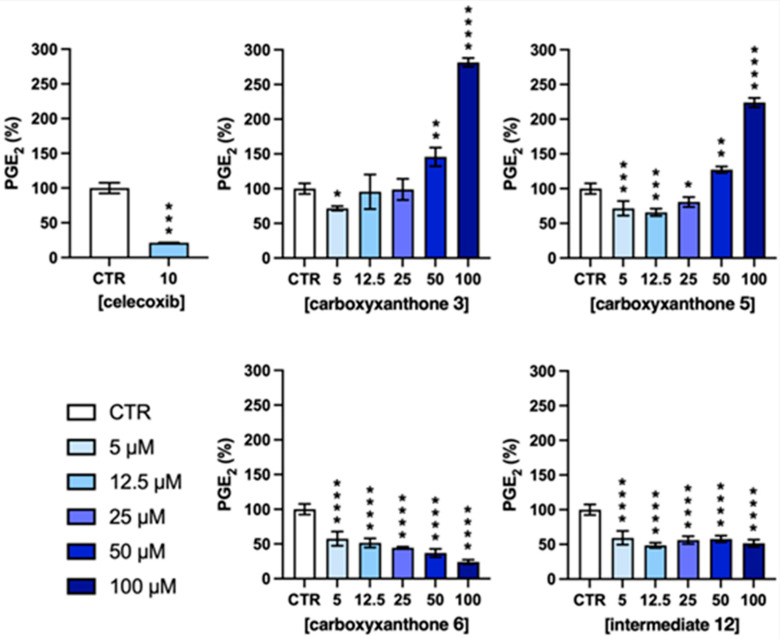
PGE_2_ production in LPS-stimulated macrophages following treatment with celecoxib, carboxyxanthones **3**, **5**, and **6**, and intermediate **12** across the indicated concentrations. PGE_2_ levels were normalized to total DNA content and expressed as a percentage relative to the LPS + vehicle control (CTR). Data were obtained from experiments performed with three independent biological samples, each analyzed in technical triplicates (total of 9 measurements per condition). Results are presented as mean ± SD. Statistical comparisons were performed using one-way ANOVA, with Dunnett’s post hoc test applied to compare each treatment group directly to the CTR group. Significance levels are indicated as: **** *p* < 0.0001, *** *p* < 0.001, ** *p* < 0.01, * *p* < 0.05. The corresponding full numeric data (means ± SDs) and sample sizes (n) are provided in Table S2.

Celecoxib, a selective COX-2 inhibitor, was used at 10 µM as a standard reference treatment to benchmark the efficacy of the tested compounds. As expected, it significantly reduced PGE_2_ production (Figure 7). Among the tested compounds, carboxyxanthone **6** exhibited the most potent and consistent inhibitory effect on PGE_2_ production, showing a clear dose-dependent response. At the highest concentration tested (100 µM), carboxyxanthone **6** approached the inhibitory efficacy observed with celecoxib, suggesting strong COX-2 modulating activity.

The intermediate **12** also led to a reduction in PGE_2_ levels, albeit with a lower potency compared to carboxyxanthone **6**. Interestingly, its inhibitory effect did not vary significantly across the tested concentrations, suggesting a plateau in activity. In contrast, carboxyxanthones **3** and **5** demonstrated a biphasic effect. At lower concentrations (5, 12.5, and 25 µM), both compounds showed little to no impact on PGE_2_ production in LPS-stimulated macrophages. However, at higher concentrations (50 and 100 µM), PGE_2_ levels increased relative to the control, suggesting a possible loss of inhibitory specificity or the activation of compensatory pro-inflammatory mechanisms. Such biphasic profiles are well documented in pharmacological studies and often reflect complex cellular feedback responses or off-target effects rather than a simple COX-2 inhibition. One plausible mechanism is the activation of a positive-feedback loop [50] whereby partial suppression of COX activity leads to compensatory upregulation of COX-2 and/or mPGES-1 expression [51]. This ultimately results in rebound PGE_2_ overproduction, a phenomenon previously described in macrophages [52]. Alternatively, higher compound concentrations could elicit cellular stress (e.g., oxidative stress) that stimulates inflammatory signaling and enhances prostanoid synthesis. Indeed, redox imbalance is known to modulate COX activity and drive increased prostaglandin production [53,54,55]. Additional off-target interactions (e.g., phospholipase A_2_ [56], lipoxygenases [57], or PGE_2_ receptor-mediated pathways [58]) may also contribute to the elevated PGE_2_ levels. Considering these possibilities, future studies should assess COX-2 and mPGES-1 expression after treatment, evaluate oxidative stress markers (e.g., ROS levels, GSH/GSSG ratio, or lipid peroxidation products, such as MDA or 4-HNE), and explore alternative eicosanoid or prostanoid pathways, including 5-LOX/12-LOX activity, cytochrome P450-derived EETs, COX-1-dependent prostanoids, or differential regulation of PGD, PGF, or thromboxane synthases.

## 3. Methods and Materials

### 3.1. Synthesis

#### 3.1.1. General

All reagents and solvents were commercially available materials *at pro analysis*, from Sigma-Aldrich Co. (St. Louis, MO, USA) or Merck (Darmstadt, Germany), and were used without further purification. The solvents were evaporated on a rotary evaporator under reduced pressure (rotative evaporator Büchi). TLC was performed using Merck silica gel 60 (GF_254_) plates, with appropriate mobile phases, and detection at 254 and/or 365 nm. Flash column chromatography was carried out using Merck silica gel 60 (0.040–0.063 mm). ^1^H and ^13^C NMR spectra were recorded in CDCl_3_ or DMSO-d_6_ at room temperature on a Bruker Avance 400 spectrometer (400 MHz for ^1^H and 101 MHz for ^13^C NMR, Bruker Biosciences Corporation, Billerica, MA, USA), at the Materials Center of the University of Porto (CEMUP), or on a Brucker DRX-300 spectrometer in the Department of Chemistry of the University of Aveiro. Chemical shifts are expressed in δ (ppm) values relative to tetramethylsilane (TMS) as an internal reference. Coupling constants are reported in hertz (Hz). Melting points were determined on a Kofler microscope (Wagner & Munz GmbH, Munich, Germany) and are uncorrected. IR spectra were obtained on a FTIR spectrometer Nicolet iS10 from Thermo Scientific (Waltham, MA, USA) with Smart OMNI-Transmission accessory (Software 188 OMNIC 8.3) in KBr. MS spectra were recorded in electronic impact mode on a VG Autospec Q spectrometer (*m*/*z*) (VG Instruments, Kuala Lumpur, Malaysia). High-resolution mass spectroscopy (HRMS) spectra were measured on a LTQ Orbitrap XL hybrid mass spectrometer (Thermo Fischer Scientific, Bremen, Germany) at CEMUP or in a Bruker Daltonics micrOTOF Mass Spectrometer, recorded in electrospray mode in Centro de Apoio Científico e Tecnológico á Investigation (C.A.C.T.I.), University of Vigo, Spain.

#### 3.1.2. Synthesis of the carboxyxanthones 6-methoxy-9-oxo-9*H*-xanthene-2-carboxylic acid (**1**) and 8-methoxy-9-oxo-9*H*-xanthene-2-carboxylic acid (**2**), and intermediate dimethyl 4-(3-methoxyphenoxy)isophthalate (**11**)

Carboxyxanthones **1** and **2** and intermediate **11** were obtained and characterized as described elsewhere [22].

#### 3.1.3. Synthesis of the carboxyxanthone 6,8-dimethyl-9-oxo-9*H*-xanthene-2-carboxylic acid (**3**)

The synthetic route used to synthesize carboxyxanthone **3** involved a multi-step pathway and is outlined in Scheme 1.

##### Synthesis of dimethyl 4-bromoisophthalate (**14**)

We added 11 mL of H_2_SO_4_ to a solution of 4-bromoisophthalic acid (**13**) (15.00 g, 61.21 mmol) in MeOH (560 mL). Then, the reaction mixture was refluxed for 17 h. After the evaporation of the MeOH, water (92 mL) was added, and the crude product was extracted with diethyl ether (3 × 100 mL). The organic layer was washed with water (100 mL), saturated NaHCO_3_ solution (3 × 100 mL), and water (2 × 100 mL). After drying the organic phase with anhydrous sodium sulfate and filtering, the solvent was evaporated under reduced pressure, affording the dimethyl 4-bromoisophthalate (**14**) as a white solid. Yield: 84%.

m.p.: 56–58 °C; IR ν_max_ (cm^−1^) (KBr): 1754, 1309, 1253, 929, 565; ^1^H NMR (CDCl_3_, 300.13 MHz) δ: 8.43 (1H, *d*, *J* = 2.2 Hz, H-2), 7.95 (1H, *dd*, *J* = 8.3 and 2.2 Hz, H-6), 7.75 (1H, *d*, *J* = 8.3 Hz, H-5), 3.95 (3H, *s*, C(1″)OOCH_3_), 3.93 (3H, *s*, C(1′)OOCH_3_); ^13^C NMR (CDCl_3_, 75.47 MHz) δ: 165.7 (C-1″), 165.5 (C-1′), 134.7 (C-4), 133.0 (C-6), 132.3 (C-2), 132.2 (C-5), 129.3 (C-3), 127.0 (C-1), 52.7 (C(1″)OOCH_3_), 52.5 (C(1′)OOCH_3_); MS (EI) *m*/*z* (%): 273 [M]^+^. (100), 256 (9), 240 (13), 221 (6), 209 (8), 203 (11).

Chromatographic monitoring was carried out by TLC using ethyl acetate:n-hexane:acetic acid (8:2:0.1 *v*/*v*/*v*) as mobile phase.

##### Synthesis of dimethyl 4-(3,5-dimethylphenoxy)isophthalate (**21**)

A mixture of dimethyl 4-bromoisophthalate (**14**) (1.01 g, 3.70 mmol), 3,5-dimethylphenol (**17**) (0.56 g, 4.60 mmol), CuI (0.04 g, 0.19 mmol), K_3_PO_4_ (1.61 g, 7.59 mmol), picolinic acid (0.05 g, 0.38 mmol) and dimethyl sulfoxide (10 mL) was heated in a sealed flask, at 80 °C, under nitrogen atmosphere, for 28 h. The reaction was filtered and then extracted with ethyl acetate (3 × 40 mL) and chloroform (3 × 50 mL). The organic phases were washed with water (3 × 50 mL), dried with anhydrous sodium sulfate, and filtered. The solvent was evaporated under reduced pressure, affording the dimethyl 4-(3,5-dimethylphenoxy)isophthalate (**21**) as a brown oil. Yield: 80%.

IR ν_max_ (cm^−1^) (KBr): 1750, 1614, 1489, 1240, 769, 700; ^1^H NMR (DMSO-d_6_, 300.13 MHz) δ: 8.36 (1H, *dd*, *J* = 2.3 and 2.0 Hz, H-2), 8.04 (1H, *dd*, *J* = 8.7 and 2.2 Hz, H-6), 6.96 (1H, *dd*, *J* = 8.7 and 2.0 Hz, H-5), 6.86 (1H, *d*, *J* = 3.4 Hz, H-4′), 6.68 (2H, *d*, *J* = 3.5 Hz, H-2′ and H-6′), 3.86 (3H, *s*, C(1′)OOCH_3_), 3.80 (3H, *s*, C(1″)OOCH_3_), 2.26 (6H, *s*, Ar-CH_3_); ^13^C NMR (DMSO-d_6_, 75.47 MHz) δ: 167.2 (C(1′)OOCH_3_), 167.1 (C(1″)OOCH_3_), 166.2 (C-3), 165.8 (C-1), 160.1 (C-4), 135.6 (C-6), 133.4 (C-2), 126.8 (C-4′), 122.3 (C-1′), 119.8 (C-5), 117.3 (C-2′ and C-5′), 113.6 (C-3′ and C-5′), 53.2 (C(1′)OOCH_3_), 53.1 (C(1″)OOCH_3_), 21.5 (Ar-CH_3_).

Chromatographic monitoring was carried out by TLC using petroleum ether:diethyl ether:acetic acid (5:5:0.1 *v*/*v*/*v*) as mobile phase.

##### Synthesis of 4-(3,5-dimethylyphenoxy)isophthalic acid (**25**)

Dimethyl 4-(3,5-dimethylphenoxy)isophthalate (**21**) (6.26 g, 19.91 mmol) was dissolved in MeOH/THF (1:1 *v*/*v*) (160 mL) and stirred at room temperature with 5 M NaOH solution (22 mL) for 18 h. After evaporation of the organic solvent, water was added (80 mL), and the crude product was washed with dichloromethane (2 × 50 mL). The organic layer was extracted with water (2 × 50 mL). The aqueous layer was acidified with 5 M HCl solution, resulting in the formation of a gray precipitate. It was collected by filtration and washed with water, to provide 4-(3,5-dimethylphenoxy)isophthalic acid (**25**). Yield: 60%.

m.p.: 198–201 °C; IR ν_max_ (cm^−1^) (KBr): 2550–3150, 1670, 1595, 1492, 1250, 770, 665; ^1^H NMR (DMSO-d_6_, 300.13 MHz) δ: 8.35 (1H, *d*, *J* = 2.3 Hz, H-2), 8.04 (1H, *dd*, *J* = 8.7 and 2.3 Hz, H-6), 6.95 (1H, *d*, *J* = 8.6 Hz, H-5), 6.84 (1H, *br s*, H-4′), 6.66 (2H, *br s*, H-2′ and H-6′), 2.26 (6H, *s*, Ar-CH_3_); ^13^C NMR (DMSO-d_6_, 75.47 MHz) δ: 166.2 (C-1′), 166.0 (C1″), 159.5 (C-3), 155.8 (C-1), 139.7 (C-4), 134.4 (C-2), 132.8 (C-6), 125.9 (C-5), 125.2 (C-4′), 123.2 (C1′), 119.1 (C-2′ and C-6′), 116.8 (C-3′and C-5′) 20.9 (Ar-CH_3_).

Chromatographic monitoring was carried out by TLC using n-hexane:ethyl acetate:formic acid (5:5:0.1 *v*/*v*/*v*) as mobile phase.

##### Intramolecular acylation: synthesis of 6,8-dimethyl-9-oxo-9*H*-xanthene-2-carboxylic acid (**3**)

To a solution of 4-(3,5-dimethylphenoxi)isophthalic acid (**25**) (0.34 g, 1.19 mmol) in methane sulfonic acid (5.5 mL) was added phosphorus pentoxide (0.56 g, 1.96 mmol). The reaction was stirred at room temperature for 48 h. The mixture was poured over ice, resulting in the formation of a dark solid that was collected by filtration and dried. The solid was crystallized from MeOH, providing 6,8-dimethyl-9-oxo-9*H*-xanthene-2-carboxylic acid (**3**). Yield: 52%.

m.p.: 191–194 °C; IR ν_max_ (cm^−1^) (KBr): 2750–3100, 1659, 1610, 1493, 1476, 1277, 770, 694; ^1^H NMR (DMSO-d_6_, 300.13 MHz) δ: 8.62 (1H, *d*, *J* = 2.1 Hz, H-1), 8.24 (1H, *dd*, *J* = 8.7 and 2.2 Hz, H-3), 7.61 (1H, *d*, *J* = 8.7 Hz, H-4), 7.25 (1H, *br s*, H-5), 7.04 (1H, *br s*, H-7), 3.17 (3H, *s*, Ar-CH_3_), 2.75 (3H, *s*, Ar-CH_3_); ^13^C NMR (DMSO-d_6_, 75.47 MHz) δ: 177.1 (C-9), 166.4 (COOH), 157.3 (C-9a), 157.0 (C-4a), 146.0 (C-10a), 141.0 (C-8a), 135.1 (C-3), 128.4 (C-1), 128.8 (C-7), 121.8 (C-6), 126.5 (C-2), 118.4 (C-4), 117.3 (C-8), 116.2 (C-5), 22.7 (C(8)CH_3_), 22.3 (C(6)CH_3_) (Figure S1, Supplementary Data). HRMS (ESI+): *m*/*z* [C_16_H_12_O_4_ + H]+ calcd. for [C_16_H_12_O_4_]: 269.08084; found 269.07919, with a calculated error of 6.11 ppm (Figure S2, Supplementary Data).

Chromatographic monitoring was carried out by TLC using dichloromethane:petroleum ether:diethyl ether:formic acid (5:5:1.5:0.1 *v*/*v*/*v*/*v*) as mobile phase.

##### Synthesis of the carboxyxanthone 5,7-dimethyl-9-oxo-9*H*-xanthene-2-carboxylic acid (**4**) and intermediate dimethyl 4-(2,4-dimethylphenoxy)isophthalate (**12**)

Carboxyxanthone **4** and intermediate **12** were obtained and characterized as described elsewhere [32].

##### Synthesis of the carboxyxanthone 7,8-dimethyl-9-oxo-9*H*-xanthene-2-carboxylic acid (**5**)

The synthetic route used to synthesize carboxyxanthone **5** involved a multi-step pathway and is outlined in Scheme 1.

##### Synthesis of dimethyl 4-(3,4-dimethylphenoxy)isophthalate (**22**)

Compound **22** was prepared from a suitable phenol, the 3,4-dimethylphenol (**19**), using the same method described for **21**. Compound **22** was obtained as a yellowish oil. Yield: 86%.

IR ν_max_ (cm^−1^) (KBr): 1750, 1610, 1480, 1240, 768, 700; ^1^H NMR (DMSO-d_6_, 300.13 MHz) δ: 8.83 (1H, *d*, *J* = 2.3 Hz, H-2), 8.09 (1H, *dd*, *J* = 8.7 and 2.3 Hz, H-6), 6.87 (1H, *d*, *J* = 8.7 Hz, H-5), 7.20 (1H, *d*, *J* = 8.5 Hz, H-5′), 6.92 (1H, *d*, *J* = 2.4 Hz, H-2′), 6.87 (1H, *dd*, *J* = 8.5 and 2.4 Hz, H-6′), 3.93 (3H, *s*, C(1′)OOCH_3_), 3.87 (3H, *s*, C(1″)OOCH_3_), 2.28 (6H, *s*, Ar-CH_3_); ^13^C NMR (DMSO-d_6_, 75.47 MHz) δ: 165.9 (C(1′)OOCH_3_), 165.6 (C(1″)OOCH_3_), 161.4 (C-4), 151.4 (C-1′), 139.4 (C-3′), 139.3 (C-6), 135.8 (C-4′), 135.3 (C-2), 134.7 (C-5′), 131.3 (C-1), 124.9 (C-6′), 121.8 (C-3), 117.9 (C-5), 116.5 (C-2′), 52.3 (COOCH_3_), 20.0 (Ar-CH_3_), 19.2 (Ar-CH_3_).

Chromatographic monitoring was carried out by TLC using petroleum ether:diethyl ether:formic acid (5:5:0.1 *v*/*v*/*v*) as mobile phase.

##### Synthesis of 4-(3,4-dimethylphenoxy)isophthalic acid (**27**)

Compound **27** was prepared using the same method previously described for compound 25. Yield: 77%.

m.p.: 156–159 °C; IR ν_max_ (cm^−1^) (KBr): 3420–2750, 1719, 1702, 1488, 1431, 1259, 765, 676; ^1^H NMR (DMSO-d_6_, 300.13 MHz) δ: 8.33 (1H, *d*, *J* = 2.9 Hz, H-2), 8.01 (1H, *dd*, *J* = 8.9 and 2.9 Hz, H-6), 6.87 (1H, *d*, *J* = 8.9 Hz, H-5), 7.18 (1H, *d*, *J* = 8.9 Hz, H-5′), 6.88 (1H, *d*, *J* = 2.9 Hz, H-2′), 6.78 (1H, *dd*, *J* = 8.9 and 2.9 Hz, H-6′), 2.21 (6H, *s*, Ar-CH_3_); ^13^C NMR (DMSO-d_6_, 75.47 MHz) δ: ^13^C NMR (DMSO-d_6_, 75.47 MHz) δ: 166.2 (C(1′)OOCH_3_), 166.1 (C(1″)OOCH_3_), 160.0 (C-4), 153.4 (C-1′), 138.5 (C-3′), 134.4 (C-6), 132.8 (C-4′), 132.5 (C-2), 130.9 (C-5′), 124.8 (C-1), 122.8 (C-6′), 120.6 (C-3), 118.2 (C-5), 116.8 (C-2′), 19.5 (Ar-CH_3_), 18.7 (Ar-CH_3_).

Chromatographic monitoring was carried out by TLC using n-hexane:ethyl acetate:formic acid (5:5:0.1 *v*/*v*/*v*) as mobile phase.

##### Intramolecular acylation: synthesis of 7,8-dimethyl-9-oxo-9*H*-xanthene-2-carboxylic acid (**5**)

Compound **5** was prepared using the same method previously described for compound **3**, but a different purification process was employed. After reaction and filtration, the solid was dissolved in methanol (400 mL), and H_2_SO_4_ (8 mL) was added. The mixture was refluxed for approximately 12 h. The products were separated by filtration, washed with cooled methanol, and purified by column chromatography (silica gel, ethyl acetate/n-hexane in gradient). The fractions containing the major amount of compound were combined. After solvent was evaporation, the resulting solid was dissolved in methanol/dichloromethane (50 mL, 1:1 *v*/*v*), and a 5 M NaOH solution (4 mL) was added. The mixture was stirred at room temperature for 22 h. After evaporation of the organic solvents, water was added (10 mL), and the solution was acidified with 5 M HCl solution, resulting in the formation of a white precipitate. The suspension was filtered under reduced pressure, and the white solid was washed with water to afford carboxyxanthone **5**. Yield: 69%.

m.p.: 256–259 °C; IR ν_max_ (cm^−1^) (KBr): 2750–3086, 1688, 1658, 1470, 1422, 1282, 770, 674; ^1^H NMR (DMSO-d_6_, 400 MHz) δ: 8.67 (1H, *d*, *J* = 2.2 Hz, H-1), 8.26 (1H, *dd*, *J* = 8.8 and 2.2 Hz, H-3), 7.64 (1H, *d*, *J* = 8.8 Hz, H-4), 7.63 (1H, *d*, *J* = 8.8 Hz, H-6), 7.39 (1H, *d*, *J* = 8.8 Hz, H-5), 2.34 (3H, *s*, Ar-CH_3_), 2.21 (3H, *s*, Ar-CH_3_); ^13^C NMR (DMSO-d_6_, 101 MHz) δ: 178.0 (C-9), 166.7 (COOH), 157.6 (C-9a), 155.8 (C-4a), 153.7 (C-10a), 139.5 (C-8), 137.1 (C-3), 135.4 (C-6), 131.3 (C-1), 128.7 (C-2), 126.6 (C-7), 119.8 (C-8a), 118.5 (C-4), 115.8 (C-5), 20.1 (C(7)CH_3_), 17.8 (C(8)CH_3_) (Figure S3, Supplementary Data). HRMS (ESI+): *m*/*z* [C_16_H_12_O_4_ + H]+ calcd. for [C_16_H_12_O_4_]: 269.08084; found 269.07981, with a calculated error of 3.81 ppm (Figure S4, Supplementary Data).

Chromatographic monitoring was carried out by TLC using ethyl acetate:n-hexane:formic acid (5:5:0.1 *v*/*v*/*v*) as mobile phase.

##### Synthesis of the carboxyxanthone 2-((9-oxo-9*H*-xanthen-3-yl)oxy)acetic acid (**6**)

Carboxyxanthone **6** was obtained and characterized as described elsewhere [31].

##### Synthesis of the carboxyxanthones 2-((9-oxo-9*H*-xanthen-1-yl)oxy)acetic acid (**7**), 2-((3-ethoxy-9-oxo-9*H*-xanthen-1-yl)oxy)acetic acid (**8**), 2,2′-((3-ethoxy-9-oxo-9*H*-xanthene-1,6-diyl)bis(oxy))diacetic acid (**9**) and 2-((8-((carboxyoxy)methyl)-3-ethoxy-9-oxo-9*H*-xanthen-1-yl)oxy)acetic acid (**10**)

Carboxyxanthones **7**, **8**, **9**, and **10** were obtained and characterized as described elsewhere [30].

### 3.2. Computational

#### 3.2.1. Preparation of Ligands and Macromolecules

The 2D structure of the small molecules was drawn using ChemDraw v20.0 (PerkinElmer Informatics, Waltham, MA, USA). The ChemBio3D v20.0 software was utilized to obtain the 3D structure of the compounds. A molecular mechanics (MM2) energy minimization was further performed to identify the most stable and least energetic molecule conformation [59]. The protein coordinates of the COX-1 and COX-2 X-ray crystal structures (PDB codes 3n8x and 1cx2, respectively) were downloaded from the Protein Data Bank of Brookhaven [60]. All structures were prepared and optimized for the docking analysis using AutoDockTools (ADT) v1.5.6 (Molecular Graphics Lab, La Jolla, CA, USA). This automated docking tool corrects the partial charges of both small molecules and target enzymes, as well as performs other chemical modifications needed for the success of the following analysis.

#### 3.2.2. Docking

The docking studies between the ligands (carboxyxanthones **1**–**10**, intermediates **11**–**12**, and positive controls) and the enzymes were carried out with the AutoDock Vina software embedded in PyRx-Virtual Screening Tool (Molecular Graphics Lab, La Jolla, CA, USA). The software considers a fixed conformation of the enzyme units, while allowing ligands to be flexible and adapt to the binding site of the respective biotarget. AutoDock Vina identified potential docking poses for the ligands, from which the ones with the highest binding affinity conformations (lowest docking score) were chosen. The software was run with an exhaustiveness of 8 and a grid box with the dimensions of X: 21.6, Y: 22.3, Z: 20.2 for COX-1; X: 22.9, Y: 23.7, Z: 21.7 for COX-2 centered on the crystallographic ligand binding site. The number of output poses was 9 per molecule. From the set of poses generated for each ligand, the most stable conformation was selected based on the lowest predicted binding free energy (ΔG, kcal/mol), which corresponds to the highest predicted affinity. This is the standard criterion used in Vina-based virtual screening and was applied consistently across all compounds. The visual inspection and graphical representation of the docking results were established with Pymol v2.3.4 (Schrödinger, New York, NY, USA) [61], as well as the identification of the hydrogen bonds, bond lengths, and other interactions between the enzymes and all the ligands [62]. A redocking validation step was also carried out by docking the native crystallographic ligands into their corresponding COX-1 and COX-2 binding sites. The resulting superimposed poses are shown in Figure S5 from Supplementary Materials. RMSD values were subsequently calculated between the crystallographic poses and the corresponding docked poses for both isoforms. The calculated RMSD values were 0.940 Å for COX-1 and 0.499 Å for COX-2, supporting the reliability of the docking protocol for the subsequent analyses.

### 3.3. Biological Activity

#### 3.3.1. Reagents

Ethanol for bioassays was sourced from Thermo Fisher Scientific (Waltham, MA, USA). The Roswell Park Memorial Institute (RPMI) 1640 medium, fetal bovine serum (FBS), antibiotic/antimycotic solution, Dulbecco’s Phosphate-Buffered Saline (DPBS), and Quant-iT PicoGreen dsDNA Kit were obtained from Thermo Fisher Scientific (Waltham, MA, USA). DMSO was supplied by VWR. The alamarBlue^®^ was purchased from Bio-Rad (Hercules, CA, USA). Enzyme-linked immunosorbent assay (ELISA) kits for human IL-6 (DuoSet) and the associated Ancillary Reagent Kit 2 were acquired from R&D Systems (Minneapolis, MN, USA). LPS from *Escherichia coli* O26:B6, phorbol 12-myristate-13-acetate (PMA), and dexamethasone were purchased from Sigma. Celecoxib was obtained from abcr GmbH (Karlsruhe, Germany). The prostaglandin E2 ELISA kit was acquired from Invitrogen (Carlsbad, CA, USA). Ultra-pure water was generated using a Milli-Q^®^ Direct Water Purification System (Milli-Q Direct 16, Millipore, Burlington, MA, USA).

#### 3.3.2. Compound Stock Solutions

Stock solutions of the compounds were prepared in DMSO at 20 mM and sterilized using 0.22 µm filters. Serial dilutions were then performed in RPMI medium. The final concentrations of the tested compounds in the wells were 5 µM, 12.5 µM, 25 µM, 50 µM, and 100 µM. Carboxyxanthone **1**, due to low solubility, was assayed at 2.5 µM, 6.25 µM, 12.5 µM, 25 µM, and 50 µM. The final concentration of DMSO in all treatments, was maintained at or below 0.2%.

#### 3.3.3. Cytocompatibility and Anti-Inflammatory Activity

The cytocompatibility and anti-inflammatory activity of the compounds were evaluated according to the method reported by Vieira et al. [28]. Briefly, human leukemia monocytic cells (THP-1 cell line) were cultured at 37 °C in a humidified atmosphere with 5% CO_2_ in complete RPMI-1640 medium (cRPMI), supplemented with 2 mM L-glutamine, 10% FBS, and 1% antibiotic/antimycotic solution. Cells were seeded at a density of 5 × 10^5^ cells/well in adherent 24-well culture plates.

To induce differentiation into macrophage-like cells, THP-1 cells were treated with 100 nM PMA for 24 h. After incubation, non-adherent cells were removed by aspiration, and adherent cells were gently washed twice with warm RPMI medium. Cells were then cultured for an additional 48 h in PMA-free RPMI medium to allow reversion to a resting macrophage phenotype.

For macrophage activation, cells were stimulated with 100 ng/mL LPS in RPMI medium for 2 h. Following this activation period, 10 µL of compound solution was added to each well containing 500 µL of LPS-containing medium. Cells were incubated with the test compounds for an additional 22 h. Afterwards, the culture medium was harvested (triplicates were combined and homogenized) and stored in aliquots at −80 °C until cytokine quantification. The cells were then washed with warm sterile PBS, and metabolic activity and DNA content were subsequently measured. LPS-stimulated macrophages cultured without compounds (0 µM) were used as a positive control for IL-6 and PGE_2_ production. Celecoxib and dexamethasone, at 10 µM, were used as positive controls of PGE_2_ and IL-6 production inhibition, respectively. Negative control comprised cells cultured without LPS stimulation.

##### Cell Metabolic Activity and DNA Concentration

The metabolic activity of macrophages, whether stimulated with LPS or not, was determined using the alamarBlue assay, as previously described [28]. Results are expressed as percentages relative to the control (untreated LPS-stimulated macrophages).

DNA content was quantified using a fluorimetric dsDNA quantification kit, following the method described previously [27]. DNA levels are expressed relative to the control (untreated LPS-stimulated macrophages).

##### IL-6 and PGE_2_ Quantification

The levels of IL-6 and PGE_2_ were measured using ELISA kits, following the manufacturer’s instructions. The PGE_2_ ELISA kit (Invitrogen) has a detection range of 0.03–2 ng/mL, while the IL-6 ELISA kit (R&D Systems) has a detection range of 9.38–600 pg/mL. Obtained values were normalized to the corresponding DNA content. Results are expressed as percentages relative to the positive control.

##### Statistical Analysis

The results are expressed as mean ± standard deviation (SD) from three independent biological replicates, each analyzed in technical triplicates per condition (9 measurements per condition). Statistical analyses were performed using GraphPad Prism 8.01 (GraphPad Software, La Jolla, CA, USA). One-way ANOVA, followed by multiple comparison tests, was used to determine significant differences between groups. A *p*-value < 0.05 was considered statistically significant.

## 4. Conclusions

In this study, a series of carboxyxanthones, including two newly synthesized derivatives (**3** and **5**), was produced and they were evaluated for their anti-inflammatory potential. All compounds showed good cytocompatibility with LPS-stimulated macrophages, and several derivatives, particularly carboxyxanthones **3**, **4**, **7**, and **8**, exhibited a pronounced ability to reduce IL-6 production. Complementary molecular docking studies provided insight into the structural features governing COX-2 recognition, enabling the identification of the most promising candidates for PGE_2_ evaluation. Among these, carboxyxanthone **6** demonstrated the strongest and most consistent inhibitory effect on PGE_2_ production, achieving an efficacy at the highest tested concentration comparable to that of celecoxib. Carboxyxanthones **3** and **5** displayed biphasic effects, suggesting the concentration-dependent modulation of COX-related pathways.

Overall, these findings support the potential of carboxyxanthones as modulators of inflammatory responses and highlight their relevance as scaffolds for the development of new anti-inflammatory agents. Nonetheless, this study also presents certain limitations. The anti-inflammatory activity of carboxyxanthones was assessed using a simplified in vitro model, which cannot fully recapitulate the complexity of in vivo inflammatory processes. Consequently, additional studies will be essential to fully establish their therapeutic potential. Moving forward, structurally guided optimization, supported by SAR analyses, computational modeling, and absorption, distribution, metabolism, excretion, and toxicity (ADMET) predictions, will aim to enhance potency, selectivity, and pharmacokinetic properties. Additionally, mechanistic studies will focus on identifying molecular targets and validating pathway-specific effects using more physiologically relevant 3D in vitro models, such as organoids, or microfluidic-based inflammatory platforms.

Subsequent steps will involve preliminary pharmacokinetic assessment, toxicity profiling, and efficacy testing via established in vivo models of acute and chronic inflammation. Collectively, these efforts will help consolidate carboxyxanthones within the anti-inflammatory drug discovery pipeline and accelerate their progression toward translational development. Although the targets implicated in anti-inflammatory activity are diverse, just like the underlying mechanisms of action and the in vitro and in vivo models employed, further research into the chemistry and biology of anti-inflammatory xanthones, as well as comprehensive SAR studies, is essential to identify the key structural features governing activity and to elucidate their mechanisms of action. Overall, the field remains highly promising and scientifically challenging, with substantial therapeutic potential.

## Figures and Tables

**Table 1:** List of noncovalent polar interactions, interacting groups and amino acids, and docking scores of the top-ranked docking pose of tested compounds and controls.

Compounds	Number of Polar Interactions		Interacting Groups		Amino Acids/ Bond Length (Å)		Binding Energy Score (kcal/mol)	
	COX-1	COX-2	COX-1	COX-2	COX-1	COX-2	COX-1	COX-2
Diclofenac	1	0	OH (carboxylic acid)	-	Arg 120 (3.15)	-	−7.1	−8.3
Indomethacin	2	4	O (amide)	O (carboxylic acid)	Arg 120 (2.86)	His 90 (3.34)	−8.0	−8.3
			OH (hydroxyl)	OH (carboxylic acid)	Leu 352 (2.98)	Leu 352 (3.07)		
				O (amide)		Tyr 355 (2.61)		
				O (carboxylic acid)		Arg 513 (3.11)		
Naproxen	1	2	OH (carboxylic acid)	O (ether)	Arg 120 (3.03)	Arg 120 (3.06)	−8.2	−8.1
				OH (carboxylic acid)		Gly 526 (3.13)		
Piroxicam	3	1	O (amide)	O (sulfate)	Val 349 (3.16)	Ser 530 (2.64)	−7.1	−8.0
			O (sulfonyl)		Ile 523 (2.46)			
			O (sulfonyl)		Ala 527 (2.70)			
Celecoxib	-	4	-	O (sulfonamine)	-	His 90 (3.18)	-	−10.0
				O (sulfonamine)		His 90 (3.50)		
				N (sulfonamine)		Leu 352 (3.06)		
				O (sulfonamine)		Arg 513 (3.27)		
Valdecoxib	-	2	-	O (sulfonamine)	-	Tyr 355 (2.90)	-	−9.0
				O (isoxazole)		Tyr 385 (2.71)		
(*S*)-Ibuprofen	1	0	OH (carboxylic acid)	-	Arg 120 (3.11)	-	−7.7	−7.4
**1**	1	1	O (diaryl ether)	O (carboxylic acid)	Tyr 355 (2.91)	Arg 120 (3.26)	−8.1	−8.4
**2**	0	1	-	O (diaryl ether)	-	Ser 530 (3.41)	−8.3	−8.6
**3**	0	1	-	OH (carboxylic acid)	-	Arg 120 (3.26)	−8.4	−9.0
**4**	1	0	O (ketone)	-	Ser 530 (2.92)	-	−8.7	−8.5
**5**	2	0	O (carboxylic acid)	-	Tyr 385 (2.92)	-	−8.2	−9.0
			O (ketone)		Ser 530 (2.93)			
**6**	1	5	O (carboxylic acid)	OH (carboxylic acid)	His 90 (3.14)	His 90 (2.97)	−8.6	−9.4
				O (carboxylic acid)		His 90 (3.24)		
				O (ether)		Gln 192 (3.00)		
				O (carboxylic acid)		Ser 533 (3.49)		
				O (diaryl ether)		Tyr 355 (2.93)		
**7**	1	0	O (ketone)	-	Ser 530 (3.20)	-	−8.8	−8.7
**8**	1	6	O (ketone)	O (carboxylic acid)	Tyr 355 (3.17)	His 90 (3.10)	−8.6	−8.9
				OH (carboxylic acid)		Gln 192 (2.98)		
				OH (carboxylic acid)		Leu 352 (3.08)		
				O (ether)		Leu 352 (3.10)		
				O (carboxylic acid)		Ser 353 (3.37)		
				O (diaryl ether)		Tyr 355 (3.07)		
**9**	1	3	O (ketone)	O (ether)	Tyr 355 (3.21)	His 90 (2.73)	−7.4	−8.3
				OH (carboxylic acid)		Leu 352 (2.90)		
				O (diaryl ether)		Tyr 355 (2.74)		
**10**	5	5	OH (carboxylic acid)	O (carboxylic acid)	His 90 (2.93)	His 90 (3.07)	−6.1	−8.7
			O (ketone)	O (ether)	Arg 120 (3.06)	His 90 (3.20)		
			O (carboxylic acid)	O (carboxylic acid)	Tyr 385 (2.73)	Gln 192 (2.99)		
			O (ether)	O (carboxylic acid)	Tyr 385 (2.80)	Ser 353 (3.47)		
			O (carboxylic acid)	O (diaryl ether)	Ser 530 (3.01)	Tyr 355 (2.94)		
**11**	1	3	O (ester)	O (ester)	Arg 120 (2.77)	His 90 (3.07)	−8.8	−8.9
				O (ester)		Arg 120 (2.97)		
				O (ether)		Leu 352 (3.17)		
**12**	2	2	O (ester)	O (ester)	Arg 120 (2.80)	His 90 (3.33)	−7.6	−9.1
			O (ester)	O (ester)	Tyr 355 (3.24)	Arg 120 (2.97)		

**Table 2:** Comparison of interactions established between COX-2 amino acids with celecoxib, carboxyxanthones **3**, **5**, and **6**, and intermediate **12**.

Celecoxib	Carboxyxanthone 3	Carboxyxanthone 5	Carboxyxanthone 6	Intermediate 12
His 90	-	-	His 90	His 90
Val 116	Val 116	Val 116	-	-
Arg 120	Arg 120	Arg 120	Arg 120	Arg 120
Gln 192	-	-	Gln 192	-
Val 349	Val 349	Val 349	Val 349	Val 349
Leu 352	Leu 352	Leu 352	Leu 352	Leu 352
Ser 353	-	-	Ser 353	Ser 353
Tyr 355	Tyr 355	Tyr 355	Tyr 355	Tyr 355
-	Leu 359	Leu 359	-	-
Phe 381	-	-	-	-
				Leu 384
Tyr 385	Tyr 385	Tyr 385	-	Tyr 385
Trp 387	Trp 387	Trp 387	-	Trp 387
Arg 513 *	**-**	**-**	Arg 513 *	Arg 513 *
Ala 516 *	**-**	**-**	Ala 516 *	**-**
Ile 517	-	-	Ile 517	-
Phe 518	Phe 518	Phe 518	Phe 518	-
Val 523 *	Val 523 *	Val 523 *	Val 523 *	Val 523 *
Gly 526	-	Gly 526	-	Gly 526
Ala 527	Ala 527	Ala 527	Ala 527	527
Ser 530	Ser 530	Ser 530	-	Ser 530
Leu 531	Leu 531	Leu 531	Leu 531	Leu 531

* The amino acids in bold are residues exclusive of COX-2. The diverse interactions are highlighted in different colors. 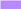
—H-bond; 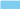
—Permanent dipole interaction; 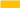
—Hydrophobic interaction.

## Data Availability

The original contributions presented in this study are included in the article/Supplementary Materials. Further inquiries can be directed to the corresponding authors.

## Supplementary Materials

The following supporting information can be downloaded at: https://www.mdpi.com/article/10.3390/ijms27010110/s1.

## Abbreviations

The following abbreviations are used in this manuscript.

ADMET	Absorption, Distribution, Metabolism, Excretion, Toxicity
bDMARDs	Biologic DMARDs
COX	Cyclooxigenase
csDMARDs	Conventional synthetic disease-modifying antirheumatic drugs
DMSO	Dimethyl sulfoxide
EMA	European Medicines Agency
FDA	Food and Drug Administration
IL-6	Interleukin 6
LPS	Lipopolysaccharide
MCP-1	Monocyte chemoattractant protein
NF-κB	Nuclear factor kappa B
NSAIDs	Nonsteroidal anti-inflammatory drugs
PGE_2_	Prostaglandin E2
RNS	Reactive Nitrogen Species
ROS	Reactive Oxigen Species
SAR	Structure-activity relationship
TNFα	Tumor necrosis factor α
tsDMARDs	Targeted synthetic DMARDs
TXA2	Thromboxane
