# Supplementary material for: Evaluation of Cytocompatibility and Anti-Inflammatory Activity of Carboxyxanthones Selected by In Silico Studies
Source: Int J Mol Sci. 2025 Dec 22;27(1):110. doi: 10.3390/ijms27010110 (PMC12785596; doi:10.3390/ijms27010110)
Supplement: Supplementary file 1 [file ijms-27-00110-s001.zip › ijms-4015307-supplementary.pdf]

Supplementary Data

Evaluation of Cytocompatibility and Anti-Inflammatory Activity of Carboxyxanthones Selected by In Silico Studies

Ricardo F. Pereira [1,†], Catarina Amoedo-Leite [1,†], Sara Gimondi [2,3], Sara F. Vieira [2,3], João Handel [1], Andreia Palmeira [1,4], Maria Elizabeth Tiritan [1,4,5,6], Madalena M. M. Pinto [1,4], Nuno M. Neves [2,3], Helena Ferreira [2,3,*] and Carla Fernandes [1,4,*]

[1] Laboratório de Química Orgânica e Farmacêutica, Departamento de Ciências Químicas, Faculdade de Farmácia, Universidade do Porto, Rua Jorge Viterbo Ferreira nº 228, 4050-313 Porto, Portugal; ricardojose.fariapereira@unifi.it (R.F.P.); catarina.leite@mcb.uu.se (C.A.-L.); up201107677@edu.ff.up.pt (J.H.); apalmeira@ff.up.pt (A.P.); beth@ff.up.pt (M.E.T.); madalenakijjoa@gmail.com (M.M.M.P.)

[2] 3B's Research Group, I3BS—Research Institute on Biomaterials, Biodegradables and Biomimetics, University of Minho, Headquarters of the European Institute of Excellence on Tissue Engineering and Regenerative Medicine, AvePark, Parque de Ciência e Tecnologia, Rua Ave 1, Edifício 1 (Sede), Barco, 4805-694 Guimarães, Portugal; sara.gimondi@i3bs.uminho.pt (S.G.); sara.vieira@i3bs.uminho.pt (S.F.V.); nuno@i3bs.uminho.pt (N.M.N.)

[3] ICVS/3B's, PT Government Associate Laboratory, 4806-909 Braga/Guimarães, Portugal

[4] CIIMAR/CIMAR LA, Interdisciplinary Centre of Marine and Environmental Research, Universidade do Porto, Terminal de Cruzeiros do Porto de Leixões, Avenida General Norton de Matos, s/n, 4450-208 Matosinhos, Portugal

[5] UCIBIO, Applied Molecular Biosciences Unit, Translational Toxicology Research Laboratory, University Institute of Health Sciences (1H-TOXRUN, IUCS-CESPU), 4585-116 Gandra, Portugal

[6] Associate Laboratory i4HB—Institute for Health and Bioeconomy, University Institute of Health Sciences—CESPU, 4585-116 Gandra, Portugal

* Correspondence: helenaferreira@i3bs.uminho.pt (H.F.); cfernandes@ff.up.pt (C.F.)

† These authors contributed equally to this work.

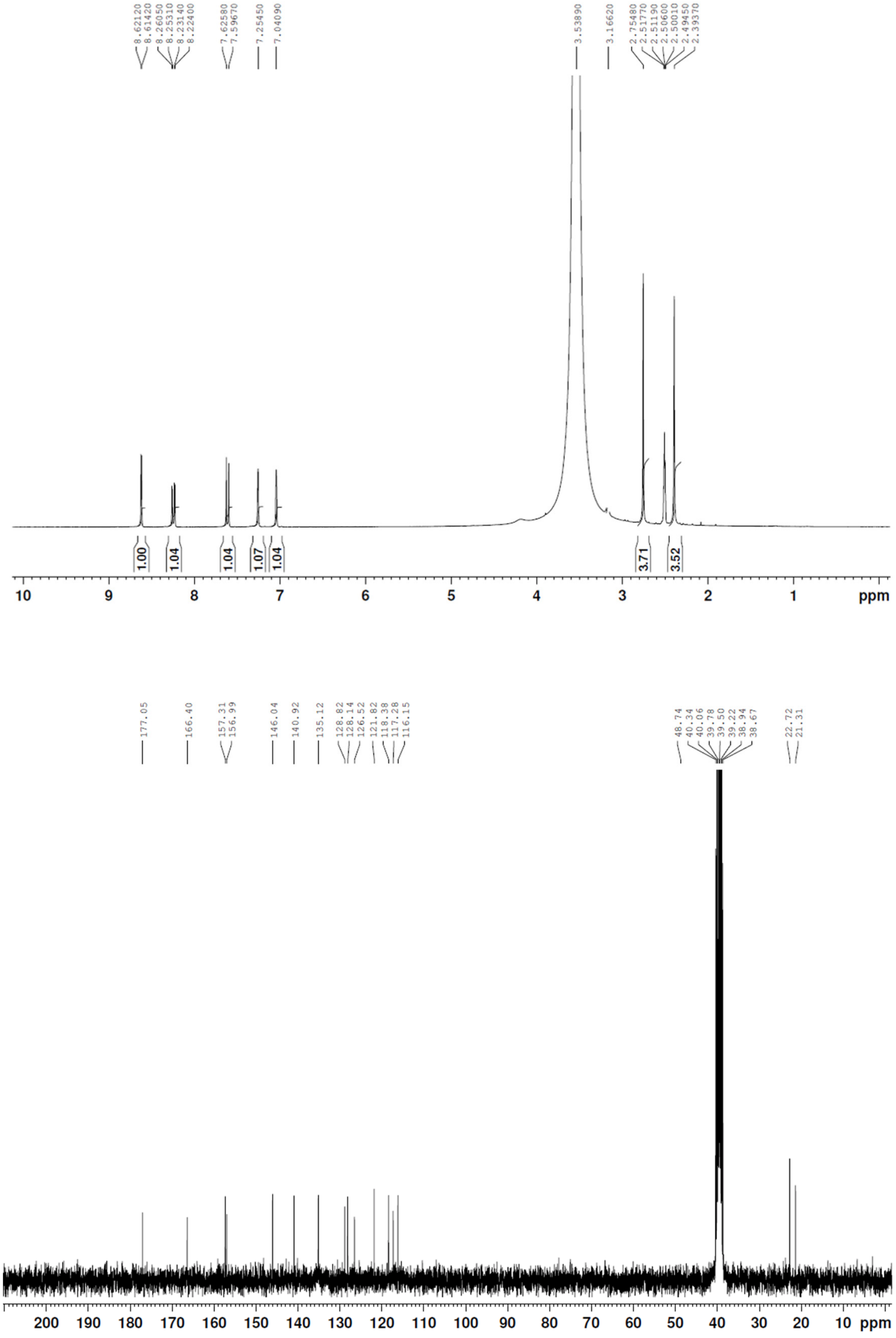

Figure S1. ^{1}H NMR (300.13 MHz, DMSO-*d6*) and ^{13}C NMR (75.48 MHz, DMSO-*d6*) for carboxyxanthone **3**.

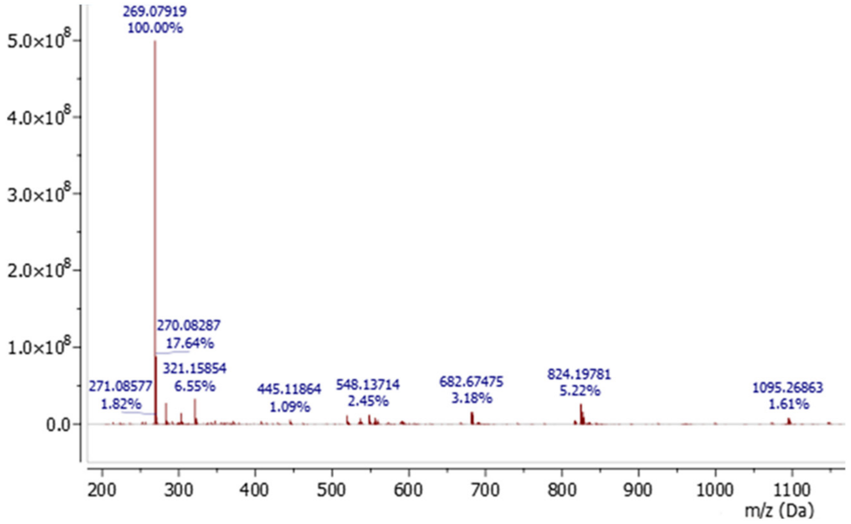

Figure S2. Electrospray ESI data for carboxyxanthone **3**.

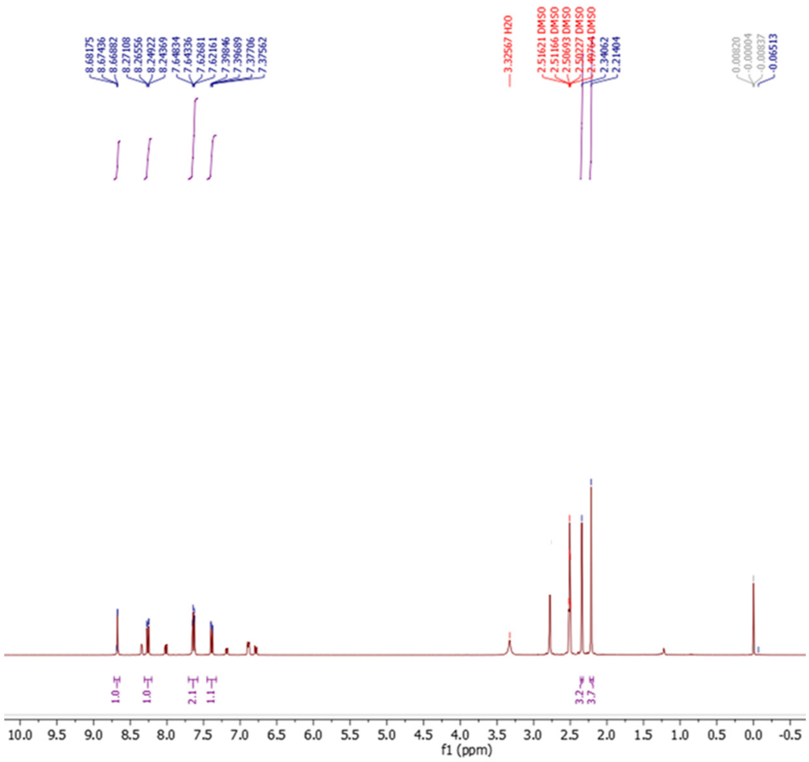

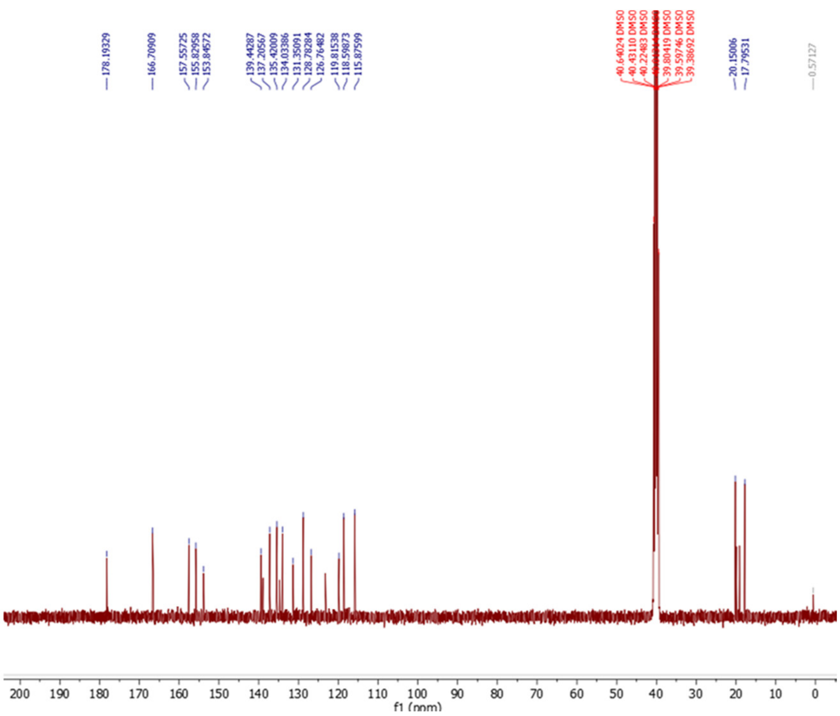

Figure S3. ^{1}H NMR (400 MHz, DMSO-*d6*) and ^{13}C NMR (101 MHz, DMSO-*d6*) for carboxyxanthone **5**.

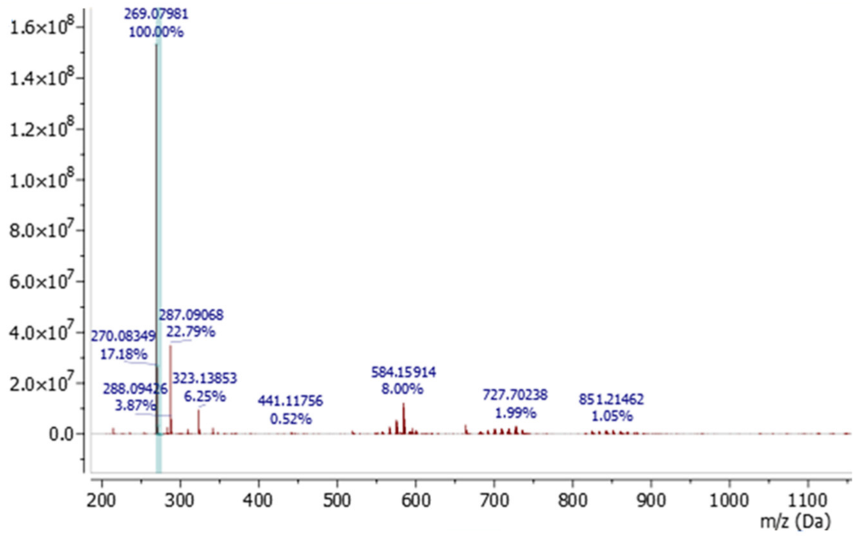

Figure S4. Electrospray ESI data for carboxyxanthone **5**.

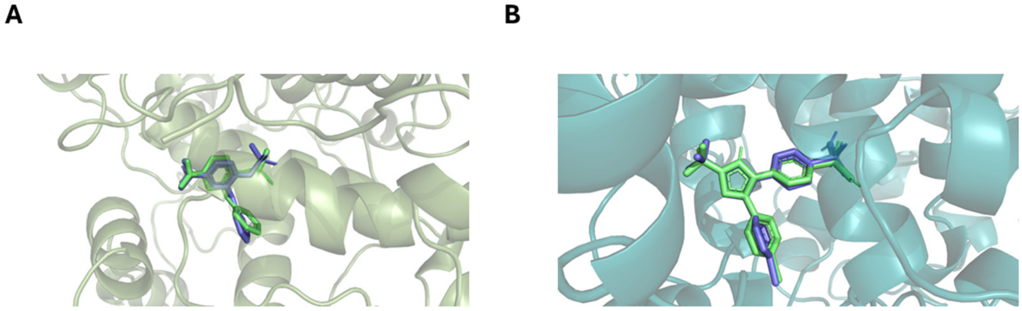

Figure S5. Superimposition of the crystallographic and docked ligands in the COX-1 (**A**) and COX-2 (**B**) binding sites. The crystallographic ligand is shown in purple, and the docked ligand is shown in green.

Table S1. Mean ± SD of % IL-6 related to control (CTR; 0 μM compound) presented in Figure 4 and number of independent biological replicates (n).

Compound	Concentration (μM)	% IL-6 related to CTR (100%; Mean ± SD)	n
**Dexamethasone**	10	10.9 ± 1.1	3
**Carboxyxanthone 1**	2.5	56.4 ± 5.3	3
	5	60.1 ± 6.7	3
	12.5	54.3 ± 3.2	3
	25	56.8 ± 7.2	3
	50	61.1 ± 5.6	3
**Carboxyxanthone 2**	5	65.1 ± 4.3	3
	12.5	77.6 ± 2.7	3
	25	52.7 ± 22.8	3
	50	61.6 ± 9.5	3
	100	74.6 ± 5.7	3
**Carboxyxanthone 3**	5	60.6 ± 13.3	3
	12.5	46.3 ± 5.2	3
	25	34.6 ± 14.7	3
	50	37.0 ± 14.5	3
	100	35.1 ± 18.6	3
**Carboxyxanthone 4**	5	46.7 ± 5.3	3
	12.5	31.2 ± 3.7	3
	25	22.5 ± 18.7	3
	50	41.1 ± 7.2	3
	100	33.4 ± 10.1	3
**Carboxyxanthone 5**	5	42.4 ± 17.7	3
	12.5	57.0 ± 16.5	3
	25	55.7 ± 13.8	3
	50	45.8 ± 25.4	3
	100	50.4 ± 25.2	3
**Carboxyxanthone 6**	5	58.0 ± 4.5	3
	12.5	44.6 ± 2.8	3
	25	49.8 ± 9.4	3
	50	57.4 ± 9.9	3
	100	70.9 ± 7.8	3

**Carboxyxanthone 7**	5	30.8 ± 2.4	3
	12.5	34.7 ± 8.9	3
	25	25.5 ± 6.4	3
	50	24.4 ± 4.2	3
	100	22.0 ± 4.2	3
**Carboxyxanthone 8**	5	69.7 ± 1.5	3
	12.5	59.8 ± 5.9	3
	25	52.5 ± 4.5	3
	50	39.0 ± 0.8	3
	100	36.8 ± 4.0	3
**Carboxyxanthone 9**	5	92.5 ± 11.2	3
	12.5	77.0 ± 8.5	3
	25	84.1 ± 12.9	3
	50	75.9 ± 1.6	3
	100	76.5 ± 1.7	3
**Carboxyxanthone 10**	5	66.1 ± 13.8	3
	12.5	60.4 ± 9.2	3
	25	67.8 ± 18.2	3
	50	69.9 ± 7.6	3
	100	60.0 ± 4.7	3
**Intermediate 11**	5	52.7 ± 19.2	3
	12.5	52.8 ± 17.2	3
	25	40.5 ±19.2	3
	50	55.7 ± 2.5	3
	100	62.3 ± 13.2	3
**Intermediate 12**	5	49.0 ± 7.1	3
	12.5	60.0 ± 4.8	3
	25	51.5 ± 6.4	3
	50	58.1 ± 1.3	3
	100	66.8 ± 1.4	3

Table S2. Mean ± SD of % PGE2 related to the control (CTR; 0 μM compound) presented in Figure 7 and number of independent biological replicates (n).

Compound	Concentration (μM)	% PGE_2 related to CTR (100%; Mean ± SD)	n
**Celecoxib**	10	21.3 ± 0.5	3
**Carboxyxanthone 3**	5	71.6 ± 3.2	3
	12.5	95.6 ± 24.9	3
	25	98.9 ± 15.2	3
	50	145.8 ± 13.4	3
	100	281.9 ± 6.3	3
**Carboxyxanthone 5**	5	71.6 ± 10.6	3
	12.5	65.9 ± 5.2	3
	25	80.8 ± 7.2	3
	50	127.3 ± 4.6	3
	100	224.0 ± 6.6	3
**Carboxyxanthone 6**	5	57.6 ± 10.3	3
	12.5	51.5 ± 6.7	3
	25	44.5 ± 1.6	3
	50	37.0 ± 5.8	3
	100	24.2 ±3.2	3
**Intermediate 12**	5	59.5 ± 9.8	3
	12.5	48.6 ± 3.7	3
	25	56.3 ± 5.6	3
	50	57.8 ± 4.7	3
	100	51.7 ± 5.2	3
